# High spatial and temporal resolution black-blood dynamic contrast-enhanced carotid artery wall MRI using compressed sensing

**DOI:** 10.1186/1532-429X-16-S1-W40

**Published:** 2014-01-16

**Authors:** Zheng-Wei Zhou, Behzad Sharif, Zhaoyang Fan, Yibin Xie, Debiao Li

**Affiliations:** 1 Biomedical Imaging Research Institute, Cedars Sinai Medical Center, Los Angeles, California, USA; 2 Department of Bioengineering, University of California, Los Angeles, Los Angeles, California, USA

## Background

Dynamic gadolinium contrast-enhanced (DCE) vessel wall imaging has been used to quantitatively assess the inflammatory status of carotid plaques[1]. However, several limitations of current techniques that may potentially compromise quantitative accuracy include: a) vessel wall blurring during high-spatial-resolution imaging due to arterial pulsation; b) inadequate separation between vessel wall and lumen; and c) relatively low temporal resolution (~20-40 sec). Recently, a black-blood DCE technique using ECG-triggering and saturation and double-inversion combined (SRDIR) preparation were proposed to overcome the first two limitations yet at the expense of temporal resolution[2]. This work aimed to accelerate the acquisition by using compressed sensing that has been applied to dynamic MRI studies[3].

## Methods

The major modification in the SRDIR DCE sequence is 2D golden-angle radial sampling in the both bright-blood and black-blood interleaf scans. This permits a retrospective selection on the temporal resolution. Image reconstruction was performed using split bregman method for l_1 _regularized optimization problems. The cost function is ||Au-b||_2_^2^+μ||▽u||_1_+γ||u-u_ref||_1_, i.e. the sum of a data fidelity term, a total variation (TV) sparsity term, and a temporal sparsity term. Volunteer data were acquired at 3T (Siemens Magnetom Verio) using a 4-channel bilateral carotid coil, with the imaging slices centered at the carotid bifurcations. Imaging parameters included: spatial resolution = 0.6 × 0.6 × 4 mm^3^, 4 slices, ECG triggering, 30 projections/R-R, 8 s/frame. Fifteen-minute continuous golden-angle radial sampling was conducted along with intravenous contrast (0.1 mmol/kg gadobenate dimeglumine) injection and saline flush (20 ml) both at 0.2 ml/s. Through ROI analysis from the bright-blood image series and dark-blood image series respectively, signal intensity change of blood pool and vessel wall was captured and used to calculate kinetic parameters.

## Results

**Figure 1:**
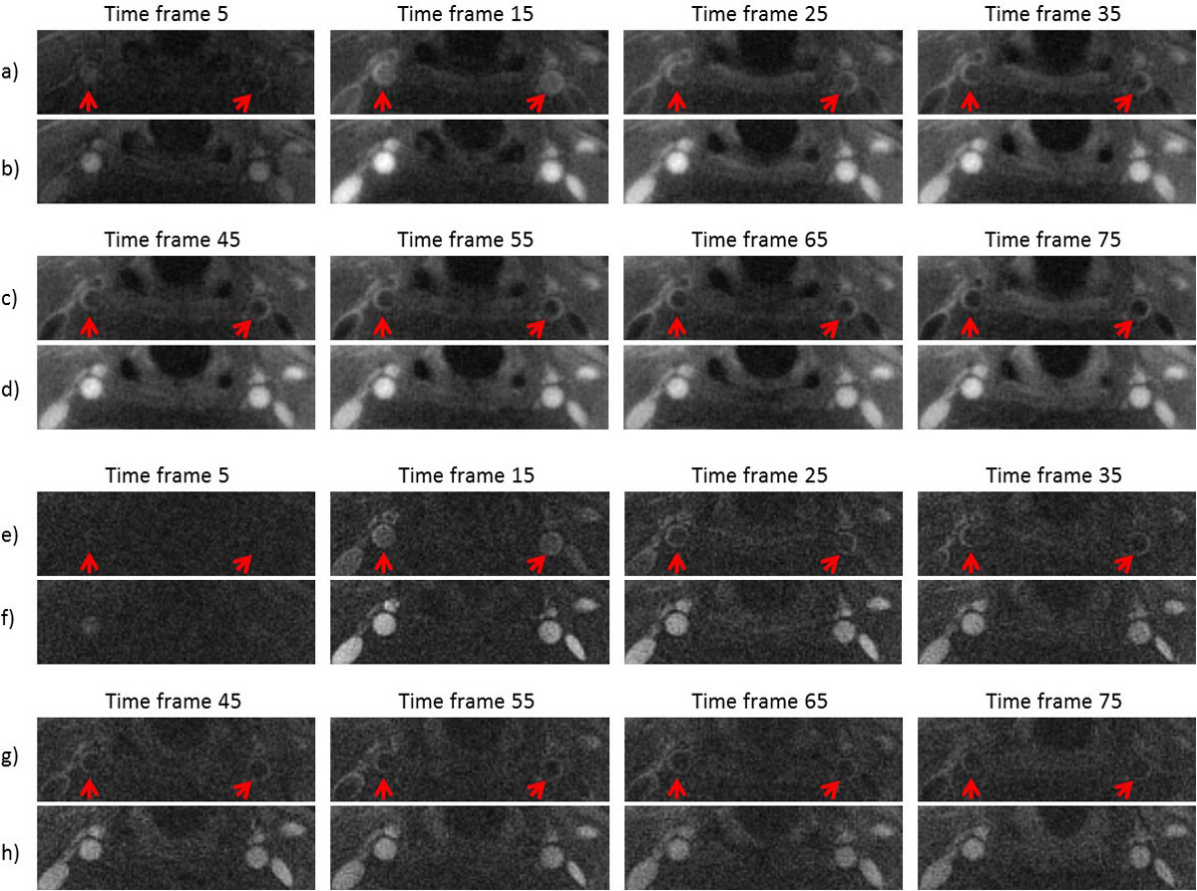
**Interleaved dark-blood (a,c) and bright-blood (b,d) images by compressed sensing compared with interleaved dark-blood (e.g) and bright-blood (f,h) images by radial regridding throughout the contrast injection process**.

**Figure 2:**
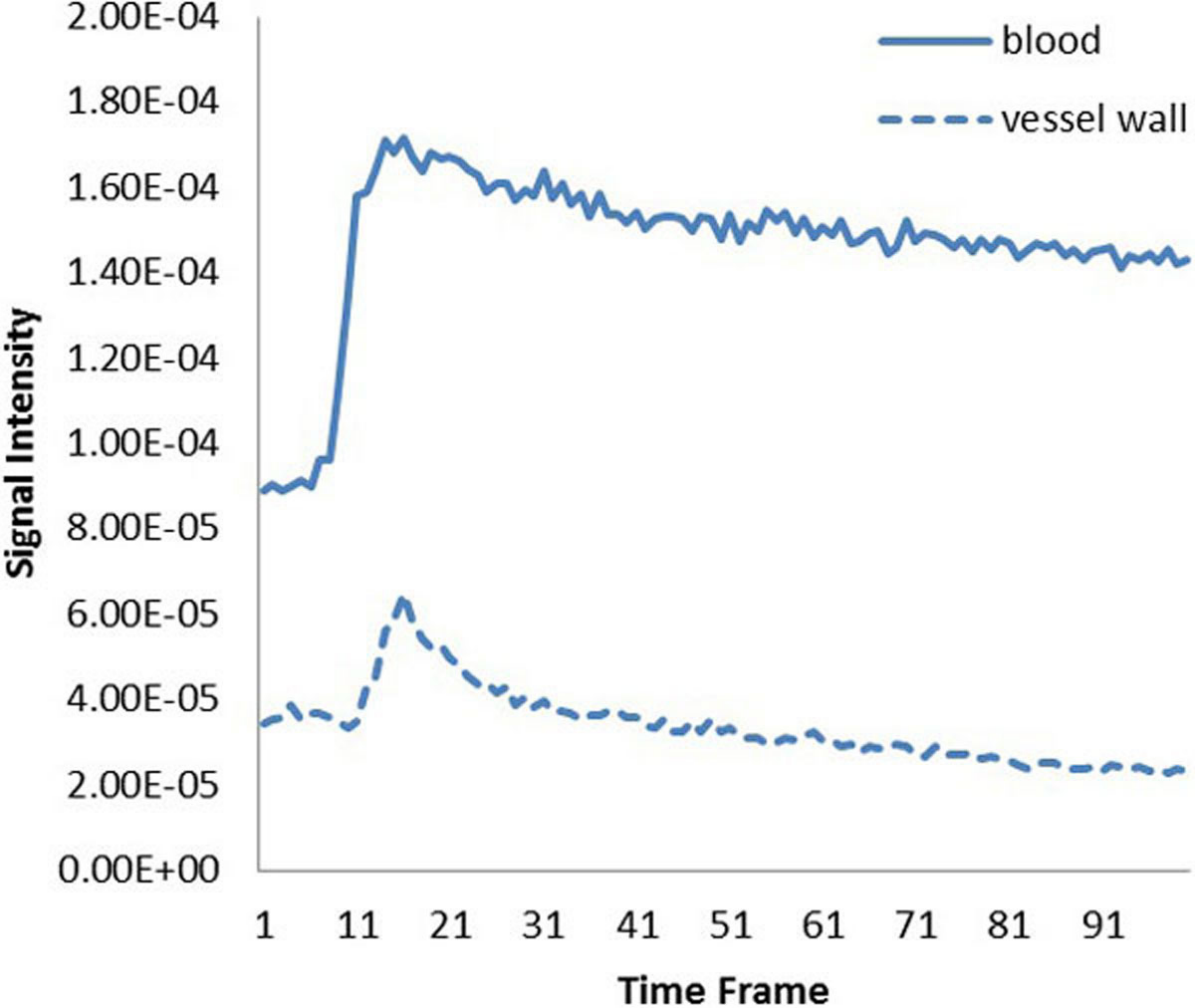
**Signal intensity vs time curves of blood and vessel wall**.

By using our new method, SNR was improved from 22.28 to 76.27 compared to conventional radial regridding method. In dark-blood image series, the vessel wall was nicely preserved (Figure 1). Because of high temporal resolution, the change in the signal intensity, especially the peak, was better captured compared to previous work (Figure 2). From this subject, K^trans ^= 0.12 min^-1^, Vp = 0.4, Kep = 0.44 min^-1^.

## Conclusions

To our knowledge, this is the first study to combine compressed sensing with black blood carotid DCE MRI. The initial data indicates that at least 2-fold temporal resolution increase is possible compared to previous carotid DCE studies by using the compressed sensing approach.

